# Methane Production on Mars-Relevant Clay Minerals and Simulant Regolith

**DOI:** 10.3390/microorganisms14071496

**Published:** 2026-07-08

**Authors:** Rebecca L. Mickol, William Hunter Waddell, James Wray, Ryan Pohlkamp, Chandler Kern, Timothy A. Kral

**Affiliations:** 1 US Naval Research Laboratory, 4555 Overlook Ave SW, Washington, DC 20375, USA; 2 Arkansas Center for Space and Planetary Science, University of Arkansas, 332 N. Arkansas Ave, Fayetteville, AR 72701, USA; 3 Department of Biological Sciences, University of Arkansas, Science and Engineering Room 601, Fayetteville, AR 72701, USA

**Keywords:** Mars, methanogens, clay minerals, montmorillonite, nontronite, illite, habitability, astrobiology

## Abstract

Over the course of Martian history, the presence of atmospheric carbon dioxide and potential subsurface molecular hydrogen (H_2_), in addition to potential surface and subsurface liquid water, suggests that the Martian subsurface, at minimum, may once have been habitable, particularly to autotrophic chemosynthetic microorganisms. In addition, the widespread nature of clays and other minerals on Mars could have provided sufficient nutrients to support microbial life. Here we tested four methanogenic species (*Methanosarcina barkeri*, *Methanobacterium formicicum*, *Methanothermobacter wolfeii*, and *Methanococcus maripaludis*) in the presence of illite, nontronite, and Mojave Mars Simulant (MMS), in their standard growth medium. We aimed to determine whether the presence of certain Mars simulants inhibited, promoted, or had no effect on methane (CH_4_) production by these microorganisms. The same methanogens were also tested in the presence of montmorillonite, H_2_, sodium sulfide (Na_2_S), and bicarbonate buffer to determine if this clay could support biotic CH_4_ production. Three of the four methanogens tested (*M. barkeri*, *M. formicicum*, and *M. wolfeii*) were capable of CH_4_ production in the presence of both clay minerals and MMS, as well as in cultures containing only 10% (*w*/*v*) montmorillonite, H_2_, Na_2_S, and bicarbonate buffer. Conversely, *M. maripaludis*, a halophile, showed the greatest sensitivity of the four methanogens tested; however, the presence of 5% (*w*/*v*) montmorillonite enabled greater CH_4_ production under certain circumstances compared to cultures containing the organism’s standard growth medium alone. Overall, these results suggest that the presence of clay minerals on Mars does not preclude the survivability and growth of methanogens in a potential subsurface habitat. In fact, these geological components may provide sufficient nutrients to support microbial growth and survivability.

## 1. Introduction

Continued exploration of the Martian surface, near-subsurface, and atmosphere have served to provide increasing amounts of evidence that Mars may once have been habitable, or may even still be habitable today, at least to microbial life. Reports of methane (CH_4_) in the Martian atmosphere [1,2,3,4,5,6,7,8,9,10,11,12] would seem to support this possibility. However, it should be noted that non-detections of CH_4_ have also been reported [13,14,15,16,17,18,19,20]. Still, it is hard not to consider methanogenesis as a possible metabolism on early or extant Mars [21,22,23,24,25,26,27,28,29,30,31,32,33,34,35,36,37,38], considering carbon dioxide (CO_2_) in the atmosphere, and likely, subsurface sources of molecular hydrogen (H_2_) [25,26,37,39,40,41,42,43,44,45,46]. The further evidence of organic carbon [47,48,49,50,51] and the widespread nature of clays [52,53,54], such as nontronite [55,56,57,58,59] and montmorillonite [60,61,62,63,64,65,66,67,68,69,70,71,72], on the planet have bolstered the idea that Mars may once have hosted microbial life.

Nontronite and montmorillonite are iron (Fe)- and aluminum (Al)-containing, respectively, dioctahedral smectites, types of phyllosilicates, formed through interaction with liquid water [69,73]. On Earth, Al- and Fe-containing smectites are often created through the alteration of basalt in environments with high water/rock ratios and thus, are typically found in marine and lacustrine environments [69]. The presence of these clays on Mars is considered to have required a volume of water seven times larger than that currently held in the Martian polar caps [74]. In addition, water adsorbed to these clays is considered to be an amount comparable to that of Earth, given Mars’ smaller size [75]. The clays, and the water they may once have held or may still hold, is important to understanding the current and past habitability of Mars [69,76], considering that water is a necessity for all life on Earth as we know it [77,78,79].

On Earth, microorganisms and clay minerals are ubiquitous across the globe and interactions between the two affect both the biosphere and the geosphere. One particular aspect of clays that forms the basis of the experiments conducted here is the cycling of numerous elements, including Al, silicon (Si), magnesium (Mg), Fe, phosphorus (P), sulfur (S), carbon (C), and nitrogen (N), through interactions between clays and microorganisms [80,81]. With smectites, in particular, such as nontronite and montmorillonite, interlayers are occupied by cations, usually K^+^, Na^+^, Ca^2+^, NH_4_^+^, or H_3_O^+^. The octahedral cation is typically Al^3+^, Fe^3+^, Mg^2+^, or Fe^2+^, but can also be less frequent elements such as lithium (Li), manganese (Mn), zinc (Zn), chromium (Cr), or titanium (Ti) [69]. In these clays, high Fe content can create unique redox conditions that microorganisms, including methanogens, can utilize as electron acceptors (e.g., Fe^3+^; see Fisk and Giovannoni [77], Zhang et al. [82], Zhang et al. [83]). In addition, the changing oxidation state of Fe in the soil can increase the bioavailability of other nutrients such as K^+^, Ca^2+^, Cu^2+^, Zn^2+^, and NH_4_^+^ [80]. On Earth, the dissolution of smectites is typically controlled by bacterial metabolism under acidic conditions [80], which could be relevant to certain environments on Mars [69,84,85,86,87,88,89]. This dissolution releases Fe, Al, and Si, as well as Na^+^ and Ca^2+^, which can serve as nutrients and promote microbial absorption to the clay minerals themselves [80].

With respect to Martian habitability, evidence for a warm and wet Mars, particularly during the Noachian and early Hesperian epochs, has been provided by explanations related to the chemical weathering of clays by acidic liquids at the surface of the planet. Using experimental modeling, Qin et al. [89] suggested that the compositional stratigraphy of Mawrth Vallis, where Fe/Mg-rich clays (e.g., nontronite) underlie Al-rich clays (e.g., kaolinite, montmorillonite), can be explained by the gradual dissolution of nontronite through the hydrolysis and leaching of Si, resulting in enrichment of Fe^3+^ and Al^3+^, and the formation of kaolinite (i.e., ferrallitization). Similarly, Ye and Michalski [90] also concluded that precipitation-driven weathering occurred on Mars from the early Noachian through the late Hesperian, which could have served as a source of vital nutrients for any extant microorganisms over geological timescales. Additionally, to explain this same compositional stratigraphy on Mars (i.e., Al-rich clays overlying Fe/Mg-rich clays), Liu et al. [91] suggested that the early Martian atmosphere was largely reducing, consisting of CH_4_ and H_2_, which enabled leaching of Fe, while Klidaras et al. [92] also contended that surface weathering and subsequent mineral leaching occurred on a warm and wet early Mars. Thus, the Noachian and Hesperian periods, generally considered to have featured long-lived habitable surface environments with temperate conditions, may have enabled microbial life to arise and flourish on the planet [76,93].

Illites are potassium micas that have also been detected on Mars [65,94] and illitization (i.e., the transition of smectite to illite) can be facilitated through microbial reduction of Fe^3+^ to Fe^2+^ [69,95], although this process is reversible (see Li et al. [80] for a comprehensive review of microbe–clay interactions). Illitization can also occur abiotically, typically through diagenesis (the conversion of sediment to sedimentary rock), which is dependent on both time and depth (i.e., burial) [94,96]. On Mars, the dominance of basalt suggests that illite was formed either through diagenesis or hydrothermal activity; however, illite is less widespread or common than either montmorillonite or nontronite, and cannot be distinguished from muscovite, another potassium mica, through spectral data alone [65,94]. In terms of the potential availability of nutrients for microbial growth and maintenance, illite (and muscovite) typically contain Al in the octahedral and tetrahedral layers, with K occupying the interlayer [67]. In contrast to smectites, illites have non-expandable basal spacing, a property which affects swelling capacity (for example, the amount of water in the interlayer), as well as molecule adsorption, including CH_4_ [80,97]. The basal spacing of illites is ca. 10 Å and lower, which could correspond to Martian data indicating similar basal spacing [95], although most interpretations believe basal spacings of 10 Å and lower to be due to collapsed smectites (due to dehydration) [56,98,99,100]. Ultimately, the widespread presence of these clays on Mars indicates significant aqueous alteration and serves to promote the habitability of clay-rich environments on the planet [52,53,54,58,59,61,62,64,65,67,68,69,70,71].

The multiple detections of CH_4_ in the Martian atmosphere from Earth-based telescopes [4,11,12], Martian orbiters [5,6,7,8,9], and Martian landers [1,2,3,10] all provide convincing evidence for extinct or extant methanogenesis on the planet. On Earth, over 80% of atmospheric CH_4_ results from the biosphere [101], including CH_4_ produced by the metabolism of microorganisms from the domain Archaea, known as methanogens. Certain methanogens are capable of using CO_2_ as a carbon source and H_2_ as an energy source to produce CH_4_ [102]. Methanogens have been considered model microorganisms for past or present life on Mars for over 20 years [23,25,103,104,105,106]. While today there are many (potentially) biocidal or inhibitory factors that would affect the ability of life to survive and thrive on the surface of the planet, such as radiation [107,108] or the lack of stable liquid water [109], among others [110,111,112], the non-photosynthetic nature of methanogens enables them to potentially persist in a subsurface habitat [22,24,25,26,28,29,33,36,102,113,114,115,116,117,118,119,120]. Additionally, their ability to use inorganic carbon (i.e., CO_2_) for their metabolism removes the constraint that the presence of other forms of life is necessary for their survival.

Research over the past 22 years has focused on various environmental and/or physiological conditions that any extant methanogens on Mars may endure, such as desiccation [106], clays and Martian regolith simulants [105,121,122,123,124], perchlorates [125,126,127], carbonates [128,129], radiation [130,131], low temperatures [103,132,133], low pressures [115,116,120], high pressures [117,118], or combinations of these conditions [104,134,135,136,137,138,139,140]. While many of these factors have been tested individually, overall results indicate that these factors may not prohibit the past or present existence of microbial life on Mars [141], and that particular niches with increased habitability, such as the subsurface [22,24,25,26,28,29,33,45,77,113,114,119,120,142,143,144,145,146,147,148], may provide sufficient conditions for microbial life to develop and perhaps, still exist, on the planet.

Here we tested the ability of four methanogenic species to grow in the presence of two common clays found on Mars, nontronite and illite, and the Martian regolith simulant, Mojave Mars Simulant (MMS) [149], in their respective growth media. For simplicity, henceforth, the clays and MMS will be referred to as Mars simulants. We aimed to determine if any of these Mars simulants were inhibitory, had no effect, or were possibly stimulatory to growth, as measured by CH_4_ production. Previous research has determined that these same methanogens are capable of growth in the presence of the Martian regolith simulant, JSC Mars-1 [105,122,123], the igneous rock, basalt [124], and the clay, montmorillonite [122,123].

Another major goal of these experiments was to determine if these same methanogens could derive their nutrient requirements (other than water, CO_2_, H_2_, and Na_2_S) from montmorillonite. Both Chastain and Kral [123] and Sinha and Kral [122] previously determined that montmorillonite could support the growth of *Methanosarcina barkeri*, *Methanobacterium formicicum*, and *Methanothermobacter wolfeii* when cells were washed to remove the residual growth medium prior to inoculation. However, these experiments included only a single washing of cells, resulting in concern that carry-over of residual nutrients from the original growth media might have allowed for growth of the methanogens. Here we report on CH_4_ production following two additional transfers in order to rule out this possibility. Lastly, an experiment was performed assessing the growth medium for the halophile, *Methanococcus maripaludis*, and whether montmorillonite could provide sufficient nutrients in place of certain medium components.

## 2. Materials and Methods

### 2.1. Cultures and Growth Media

Methanogens were initially obtained from the Oregon Collection of Methanogens (OCM, Portland State University, Portland, OR, USA) or the American Type Culture Collection (ATCC, Manassas, VA, USA). Each methanogen was grown in its own anaerobic medium and at its own growth temperature: *Methanosarcina barkeri* (OCM 38, ATCC 43569), 37 °C, MS medium (yeast extract, trypticase peptone, mercaptoethanesulfonic acid, potassium phosphate, ammonium chloride, magnesium chloride, calcium chloride, and additional trace minerals) [106,150]; *Methanobacterium formicicum* (OCM 55, ATCC 33274), 37 °C, MS medium supplemented with sodium formate (designated MSF medium) [150]; *Methanothermobacter wolfeii* (OCM 36, ATCC 43096), 55 °C, MM medium (a minimal medium containing the same components as MS medium except yeast extract, trypticase peptone, and mercaptoethanesulfonic acid) [106,151]; and *Methanococcus maripaludis* (OCM 151, ATCC 43000), 22 °C, MSH medium (MS medium containing additional sodium chloride, magnesium chloride, and potassium chloride) [152]. All media were created in bicarbonate buffers (4 g/L sodium hydroxide (NaOH) saturated with CO_2_). These media and temperatures were used for growth of the organisms and are not meant to mimic available nutrients or environmental conditions on Mars. Growth was measured by increases in CH_4_ production over time via gas chromatography (GC, CP-4900, Varian Micro Gas Chromatograph, Palo Alto, CA, USA) [153]. Specifically, for all experiments described here, individual 3 mL syringes were used to remove 1 mL headspace from each tube, which was subsequently injected into the GC to determine CH_4_ concentration.

Typically, CH_4_ concentrations of 0.1% or higher increasing with time [123] are considered to be an indication of growth. For clarity, here we acknowledge that while methanogens are obligate CH_4_-producers, the production of which constitutes the energy-yielding step in methanogenesis [102,154], CH_4_ measurements provided without additional biomass data may be indicative solely of maintenance metabolism and not cell division [155]. However, we also argue that any active metabolism, whether representative of cell division or maintenance metabolism, remains highly relevant to (potential) subsurface life on both Earth and Mars, with specific respect to ultra-slow-growing microorganisms termed aeonophiles [38,156]. In particular, Lloyd and Steen [156] define aeonophiles as a unique class of extremophiles that persist in a metabolically active maintenance state for years or longer. These organisms typically inhabit the terrestrial deep subsurface and could represent potential subsurface life on Mars [38,156].

### 2.2. Mars Simulants

Three clays were used in the experiments described here that are akin to those commonly identified on the surface of Mars. Nontronite, an Fe-smectite (NAu-1, Uley Mine, Uley, Australia; [157,158]), and illite, an Al-phyllosilicate (IMt-1/2, Silver Hill, MT, USA), were obtained from the Clay Minerals Society Source Clays Repository (Chantilly, VA, USA). Montmorillonite, an Al-smectite, was obtained from Ward’s Science (Rochester, NY, USA) with the clay itself originating from Clay Spur, WY, USA. Mojave Mars Simulant (MMS) [149] is a basaltic Martian simulant regolith selected for the similarity of its chemical and physical characteristics to those measured by various Martian rovers. General chemical compositions for the Mars simulants are given in Table 1.

### 2.3. Experimental Procedures

#### 2.3.1. Methane Production in the Presence of Illite, Nontronite, or Mojave Mars Simulant in Standard Growth Media

Growth experiments in the presence of illite were performed with the addition of either 1% (*w*/*v*) or 2% (*w*/*v*) illite clay to each methanogen’s respective anaerobic growth medium (see Section 2.1 Cultures and Growth Media). More specifically, growth media were prepared in flasks and transferred to a Coy anaerobic chamber (90:10 CO_2_:H_2_; Coy Laboratory Products Inc., Grass Lake Charter Township, MI, USA) to deoxygenate for 24–36 h. Either 0.1 g illite [1% (*w*/*v*)] or 0.2 g illite [2% (*w*/*v*)] were added to each of five Balch tubes (internal volume ca. 25 mL), after which 10 mL growth medium was distributed to each tube for each organism (*n* = 3–5; Table 2). This resulted in ca. 15 mL available headspace within each tube (see Appendix A for further calculations). The tubes were capped with butyl rubber stoppers and aluminum crimps and autoclaved (121 °C, 15 psi, 30 min). Afterward, ca. 0.125 mL 2.5% Na_2_S were added to each tube to remove residual oxygen. Each tube was inoculated with 0.5 mL of culture containing the respective methanogen, pressurized with 2 bar H_2_ gas, and kept at the organisms’ standard growth temperature. Tubes were monitored for CH_4_ production (measured via GC) over 29–31 days. Positive control tubes containing solely 10 mL growth medium without illite (*n* = 3–5) were also monitored for CH_4_ production over time (Table 2).

Experimental procedures for the nontronite and MMS experiments were identical to those for the illite experiments except for the amount of nontronite or MMS and medium in each tube. For the nontronite experiments, three sets of tubes were inoculated: 0 g nontronite in 10 mL medium (*n* = 3; positive control tubes), 1 g nontronite in 9 mL medium (11.1% (*w*/*v*), *n* = 3) or 2.5 g nontronite in 7.5 mL medium (33.3% (*w*/*v*), *n* = 3). Methane production was monitored via GC over 35 days (Table 2). For the MMS experiments, tubes contained 10 g MMS and 10 mL medium (100% (*w*/*v*), *n* = 3). Positive control tubes contained solely 10 mL medium (no MMS, *n* = 2). Tubes were monitored for CH_4_ production over 140 days (Table 2). Because the MMS tubes contained greater amounts of clay simulant (10 g) and liquid media (10 mL) than the other experiments conducted here, it is possible that the remaining headspace in the Balch tubes was less than that for the other experiments. In all other experiments described here, the headspace volume was ca. 15 mL; in the MMS experiments, the available headspace volume was likely closer to ca. 10 mL, which would result in fewer total moles of CH_4_ produced (see Appendix A for calculations). However, since we are most interested in increases in CH_4_ concentrations over time, whether portrayed as % headspace or as moles CH_4_, we have not performed these calculations for the data shown here (except for data provided in Table S1).

#### 2.3.2. Methane Production with Montmorillonite as the Sole Nutrient Source

Experimental procedures for the montmorillonite experiments were conducted to remove any residual growth media from the inoculated tubes, forcing the methanogens to obtain any necessary nutrients from the clay mineral itself. Cells grown in their respective anaerobic growth media were centrifuged at 5000 rpm for 15 min, the supernatant was discarded, and the pellet was resuspended in sterile bicarbonate buffer. This wash procedure was repeated two more times to rid the cells of any nutrients contained in their original respective growth medium [160]. Next, 0.5 mL aliquots of washed cells were added to Balch tubes (ca. 25 mL internal volume) containing sterile montmorillonite (1 g) and 10 mL bicarbonate buffer (10% (*w*/*v*), *n* = 3 for each organism), leaving ca. 15 mL headspace volume (see Appendix A for calculations). Next, each tube was pressurized with 2 bar H_2_ gas. The tubes were monitored for CH_4_ production over time for six weeks. In order to ensure that no residual nutrients remained in the experimental tubes following inoculation, two transfer sets were also created: at six-week intervals, a new set of tubes containing 1 g montmorillonite and 10 mL bicarbonate buffer were inoculated with 0.5 mL culture (i.e., cells in buffer with 1 g montmorillonite) from the corresponding tube in the previous set. This resulted in three transfers over 18 weeks. For clarity, Set A was inoculated with 0.5 mL washed cells and CH_4_ was measured for six weeks. At six weeks, 0.5 mL culture from Tube 1 from Set A were used to inoculate Tube 1 from Set B. Both Sets A and B were monitored for CH_4_ production for an additional six weeks. At six weeks from the time that Set B was inoculated, 0.5 mL culture from Tube 1 from Set B were used to inoculate Tube 1 from Set C and Sets A, B, and C were monitored for CH_4_ production for an additional 16 weeks (Table 3). At the creation of the new set (i.e., Set B or Set C), tubes within the previous set (i.e., Set A or Set B) were re-pressurized with H_2_. Specifically, this occurred after 42 days’, 84 days’, and 144 days’ incubation for Set A, after 42 days’ and 102 days’ incubation for Set B, and after 60 days’ incubation for Set C.

#### 2.3.3. Minimal Medium Requirements for *Methanococcus maripaludis*

Following from the inability of *M. maripaludis* to grow in cultures containing montmorillonite as the sole nutrient source (Table 3), a separate experiment was conducted to determine the minimal medium requirements for this organism and whether montmorillonite could serve as a replacement for any necessary medium components. Thus, seven variations of MSH medium with montmorillonite (Table 4) were tested to determine which medium components are vital to the growth of *M. maripaludis* and whether montmorillonite could provide certain necessary nutrients. Experimental procedures were identical to those performed for the illite, nontronite, and MMS experiments, except that tubes contained 0.5 g montmorillonite and 10 mL of each medium variation (5% (*w*/*v*), *n* = 3, Table 4). Three tubes, used as positive controls, contained MSH medium and no montmorillonite. All tubes were incubated at room temperature (ca. 22 °C) for three weeks and periodically monitored for CH_4_ production via GC.

### 2.4. Statistical Analysis

All data are represented as mean ± standard deviation. The number of biological replicates for each condition and each experiment are provided in Table 2. All statistical analyses were conducted in R (v4.2.2). One-way ANOVAs were performed with Tukey’s HSD post hoc test with a 95% family-wise confidence level for multiple comparisons. Computed *p*-values below 0.1 were considered statistically significant (ns: not significant, .: *p* < 0.1, *: *p* < 0.05, **: *p* < 0.01, ***: *p* < 0.001).

## 3. Results

### 3.1. Methane Production in the Presence of Illite, Nontronite, and Mojave Mars Simulant

**Figure 1:**
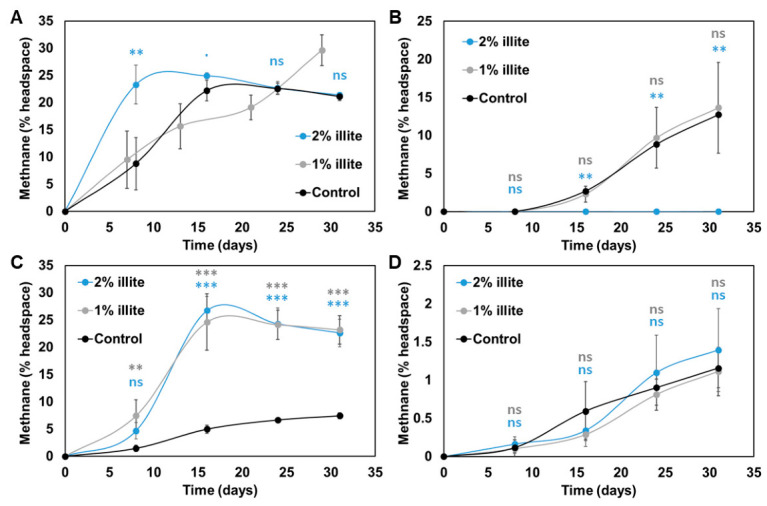
Methane production (% headspace) over time for four methanogens grown in their respective anaerobic growth medium with either 0% (control), 1% (*w*/*v*), or 2% (*w*/*v*) illite clay: (**A**) *Methanothermobacter wolfeii* (55 °C, MM medium); (**B**) *Methanococcus maripaludis* (22 °C, MSH medium); (**C**) *Methanobacterium formicicum* (37 °C, MSF medium); and (**D**) *Methanosarcina barkeri* (37 °C, MS medium). Data are the average of 3–5 replicates (Table 2). Error bars represent ± one standard deviation. Statistical differences revealed by ANOVA are presented compared to the control condition (ns: not significant, .: *p* < 0.1, **: *p* < 0.01, ***: *p* < 0.001).

When grown in the presence of 2% (*w*/*v*) illite, CH_4_ production reached a higher concentration after 8 days’ incubation for *M. wolfeii* than within control tubes (*p* < 0.01), although final CH_4_ concentrations after 32 days’ growth were not significantly different from cultures grown in the absence of illite (*p* > 0.1, Figure 1A). Methane production was similar between *M. wolfeii* cultures grown in the presence of 1% (*w*/*v*) illite or without illite (control samples); however, statistical analysis could not be completed due to differences in time point (Figure 1A). Methane concentrations reached a maximum of 29.6 ± 2.8% headspace after 29 days for *M. wolfeii* grown with 1% (*w*/*v*) illite and a maximum of 24.9 ± 0.6% headspace after 16 days with 2% (*w*/*v*) illite. *M. wolfeii* cultures grown in their standard growth medium (MM medium) without clay minerals reached a maximum CH_4_ concentration of 22.5 ± 1.0% headspace after 24 days’ incubation (Figure 1A, Table 2). *M. formicicum* showed enhanced growth (higher CH_4_ production) in the presence of the clay mineral than in standard anaerobic growth medium (Figure 1C, Table 2). More specifically, *M. formicicum* cultures reached a maximum CH_4_ concentration of 24.6 ± 5.2% headspace after 16 days in the presence of 1% (*w*/*v*) illite (*p* < 0.001, compared to control cultures) and 26.7 ± 2.6% headspace after 16 days in the presence of 2% (*w*/*v*) illite (*p* < 0.001, compared to control cultures). In comparison, cultures of *M. formicicum* grown in MSF medium without clay minerals reached a CH_4_ concentration of 7.4 ± 0.5% headspace after 31 days’ incubation (Figure 1C). For *M. barkeri*, CH_4_ production was not affected by the presence of illite with maximum CH_4_ concentrations ca. 1% headspace after 31 days’ incubation amongst all conditions with no statistically significant differences seen (*p* > 0.1, Figure 1D, Table 2). When grown with 1% (*w*/*v*) illite, *M. maripaludis* showed CH_4_ production similar to the control tubes (ca. 13% headspace after 32 days’ incubation, *p* > 0.1); however, 2% (*w*/*v*) illite proved inhibitory as no CH_4_ production occurred in these cultures (*p* < 0.01, Figure 1B, Table 2).

**Figure 2:**
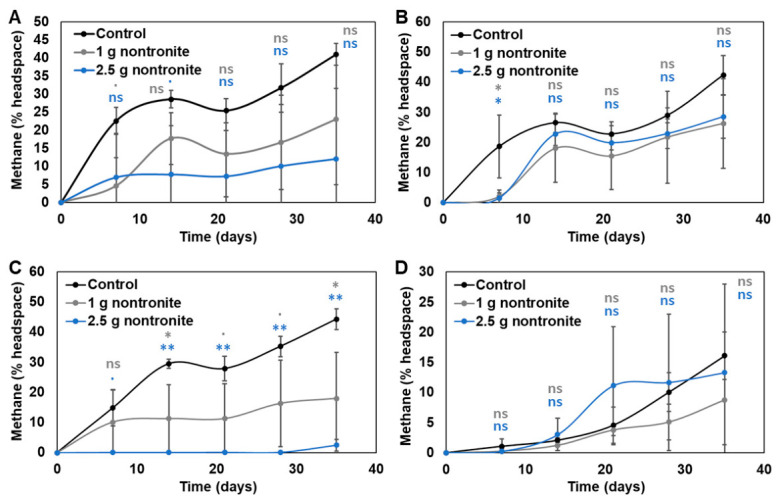
Methane production (% headspace) over time for four methanogens grown in their respective anaerobic growth medium with either 1 g (in 9 mL medium) or 2.5 g (in 7.5 mL medium) nontronite clay: (**A**) *Methanothermobacter wolfeii* (55 °C, MM medium); (**B**) *Methanococcus maripaludis* (22 °C, MSH medium); (**C**) *Methanobacterium formicicum* (37 °C, MSF medium); and (**D**) *Methanosarcina barkeri* (37 °C, MS medium). Control tubes contain only 10 mL medium. Data are the average of three biological replicates (Table 2). Error bars represent ± one standard deviation. Statistical differences revealed by ANOVA are presented compared to the control condition (ns: not significant, .: *p* < 0.1, *: *p* < 0.05, **: *p* < 0.01).

In the nontronite experiments, all four methanogens were capable of CH_4_ production in cultures containing up to 2.5 g nontronite [33.3% (*w*/*v*)], although CH_4_ production was greatly delayed and reduced for *M. formicicum* at this concentration (Figure 2C). After 35 days, CH_4_ concentrations reached 2.4 ± 2.9% headspace for *M. formicicum* grown in the presence of 2.5 g nontronite whereas cultures grown in MSF medium without clay minerals reached a maximum concentration of 44.3 ± 3.4% headspace CH_4_ (*p* < 0.01, Figure 2C, Table 2). Methane production was also significantly lower in cultures containing 1 g [11.1% (*w*/*v*)] nontronite (compared to control cultures) over the course of the experiment (*p* < 0.1, Figure 2C). Similar to *M. formicicum*, CH_4_ production decreased for cultures of *M. wolfeii* with increasing nontronite concentration: CH_4_ reached concentrations of 41.0 ± 3.0% headspace for cultures without nontronite, 23.0 ± 18.1% headspace for cultures grown with 1 g nontronite, and 12.1 ± 19.4% headspace for cultures grown with 2.5 g nontronite; however, these differences were not statistically significant after 20 days’ incubation (*p* > 0.1, Figure 2A, Table 2). For *M. maripaludis*, CH_4_ production was similar between experimental tubes containing either 1 g or 2.5 g nontronite (ca. 26–28% headspace), but was higher for cultures grown without clay minerals (42.3 ± 6.6% headspace), although these differences were not significant after 14 days’ incubation (*p* > 0.1, Figure 2B, Table 2). Methane production was not significantly different amongst any of the conditions at any of the time points for *M. barkeri* (*p* > 0.1, Figure 2D, Table 2). Notably, CH_4_ production amongst replicates for the same organism and same conditions varied greatly, resulting in large error bars (Figure 2).

**Figure 3:**
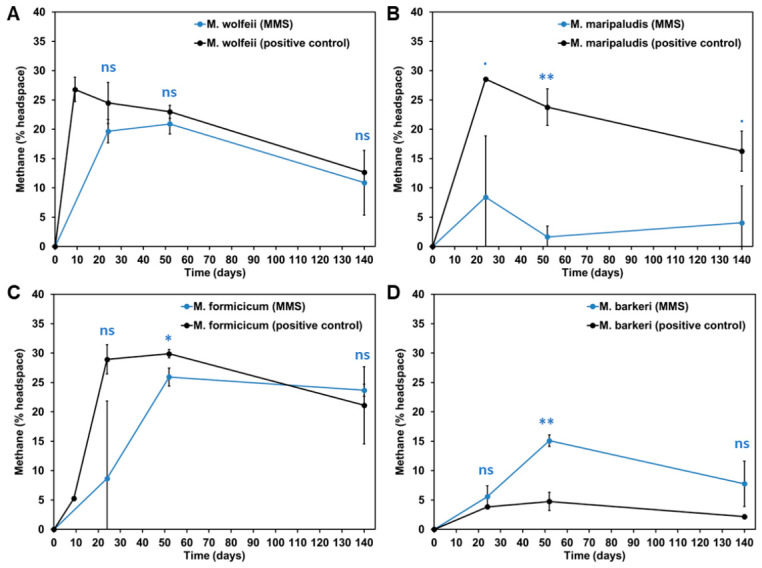
Methane production (% headspace) over time for four methanogens grown in their respective anaerobic growth medium with either 0% (positive control) or 100% (*w*/*v*) [10 g/10 mL] Mojave Mars Simulant (MMS) Martian regolith simulant: (**A**) *Methanothermobacter wolfeii* (55 °C, MM medium); (**B**) *Methanococcus maripaludis* (22 °C, MSH medium); (**C**) *Methanobacterium formicicum* (37 °C, MSF medium); and (**D**) *Methanosarcina barkeri* (37 °C, MS medium). Data are the average of either two (positive controls) or three (experimental tubes) biological replicates (Table 2). Error bars represent ± one standard deviation. Statistical differences revealed by ANOVA are presented compared to the control condition (ns: not significant, .: *p* < 0.1, *: *p* < 0.05, **: *p* < 0.01).

For *M. wolfeii* and *M. formicicum,* growth was similar in both the presence and absence of MMS, but with slightly higher CH_4_ production in tubes without MMS (Figure 3A,C, Table 2). For *M. wolfeii*, CH_4_ concentration peaked at 26.8 ± 2.1% headspace after 9 days’ incubation at 55 °C in cultures without MMS, while CH_4_ concentration reached 20.9 ± 1.7% headspace in cultures containing MMS after 52 days’ incubation; however, there were no statistically significant differences in CH_4_ concentration at 24, 52, or 140 days’ incubation (*p* > 0.1, Figure 3A, Table 2). Maximum CH_4_ concentrations for *M. formicicum* were higher in cultures without MMS (reaching 29.9 ± 0.7% headspace after 52 days’ incubation at 37 °C), while cultures containing MMS reached 25.9 ± 1.5% headspace after 52 days’ incubation (*p* > 0.05, Figure 3C, Table 2). *M. maripaludis* was the only organism where the presence of MMS significantly hindered CH_4_ production by the organism (compared to growth in standard medium). Methane production peaked at 8.4 ± 10.4% headspace after 24 days’ incubation at 22 °C for *M. maripaludis* cultures containing MMS, but CH_4_ concentrations between the three biological replicates were considerably different (20.3% vs. 4.2% vs. 0.8% headspace). In cultures without MMS, *M. maripaludis* produced 28.6 ± 0.2% headspace CH_4_ after 24 days’ incubation (*p* < 0.1, Figure 3B, Table 2). In contrast to the other three organisms, *M. barkeri* was the only methanogen that produced greater amounts of CH_4_ in the presence of MMS than in the absence of MMS. Methane production by *M. barkeri* peaked at 15.1 ± 1.0% headspace in cultures containing MMS after 52 days’ incubation, whereas cultures without MMS reached only 4.8 ± 1.6% headspace CH_4_ in the same amount of time (*p* < 0.01, Figure 3D, Table 2). However, it is important to note that there were no statistically significant differences in CH_4_ concentration between the *M. barkeri* cultures containing MMS and the cultures without MMS at either the 24-day or 140-day time points (*p* > 0.1, Figure 3D).

### 3.2. Methane Production with Montmorillonite as the Sole Nutrient Source

**Figure 4:**
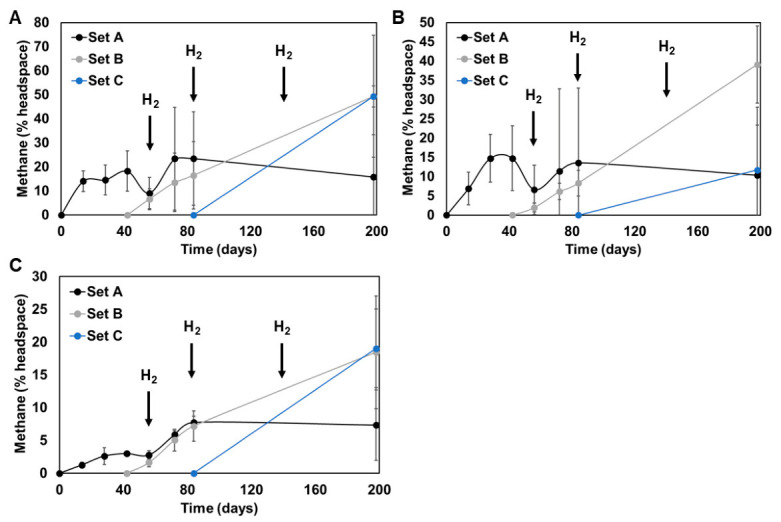
Methane production (% headspace) over time for three methanogens grown in 10 mL bicarbonate buffer with 1 g montmorillonite clay [10% (*w*/*v*)] and subject to two subsequent transfers to new tubes containing 1 g montmorillonite clay and 10 mL bicarbonate buffer: (**A**) *Methanothermobacter wolfeii* (55 °C); (**B**) *Methanobacterium formicicum* (37 °C); and (**C**) *Methanosarcina barkeri* (37 °C). Initial inocula consisted of 0.5 mL washed cells (Set A). Each tube in the subsequent sets (Set B, Set C) was inoculated with 0.5 mL culture from the corresponding tube in the preceding set (i.e., Tube B1 was inoculated with 0.5 mL culture from Tube A1). All tubes were pressurized with 2 bar H_2_ (arrows) on days 0, 42, 84, and 144 (Set A), days 42, 84, and 144 (Set B), or days 84 and 144 (Set C). Data are the average of three biological replicates (Table 3). Error bars represent ± one standard deviation.

In the montmorillonite experiments, each of the four methanogens was grown in bicarbonate buffer containing H_2_, Na_2_S, and 10% (*w*/*v*) montmorillonite (*n* = 3, Table 3). After six weeks, 0.5 mL culture was used to inoculate another tube containing 10% (*w*/*v*) montmorillonite in bicarbonate buffer. In addition, all tubes (from all sets) were pressurized with 2 bar H_2_ at 0, 42, 84, and 144 days. This pressurization at 42 days likely results in the decrease in CH_4_ as a percentage of the headspace (seen in Figure 4A–C). Data indicate that each of three methanogens (*M. wolfeii*, *M. formicicum*, and *M. barkeri*) were capable of CH_4_ production in tubes containing solely bicarbonate buffer, montmorillonite, Na_2_S, and H_2_ gas (Figure 4, Table 3). In contrast, *M. maripaludis* was incapable of any CH_4_ production. For *M. wolfeii* and *M. barkeri*, cultures in Set A reached maximum CH_4_ concentrations after 84 days’ incubation (23.5 ± 19.4% headspace and 7.8 ± 1.0% headspace, respectively; Figure 4A,C, Table 3). *M. formicicum* cultures in Set A reached a maximum CH_4_ concentration of 14.8 ± 4.0% headspace after 42 days’ incubation (Figure 4B, Table 3). For Set B cultures, CH_4_ production reached higher concentrations than Set A cultures for each methanogen after 156 days’ incubation: 49.3 ± 24.3% headspace (*M. wolfeii*), 18.5 ± 8.6% headspace (*M. barkeri*), and 39.1 ± 10.1% headspace (*M. formicicum*; Figure 4, Table 3). Methane concentrations were only measured once after 114 days’ incubation for Set C cultures. After 114 days’ incubation, CH_4_ concentrations for *M. wolfeii* and *M. barkeri* for Set C were similar to the concentrations reached for Set B (49.3 ± 4.4% headspace and 19.0 ± 6.0% headspace, respectively; Figure 4A,C, Table 3). Set C cultures for *M. formicicum* measured 11.7 ± 11.7% headspace after 114 days’ incubation (Figure 4B, Table 3).

### 3.3. Minimal Medium Requirements for Methanococcus maripaludis

**Figure 5:**
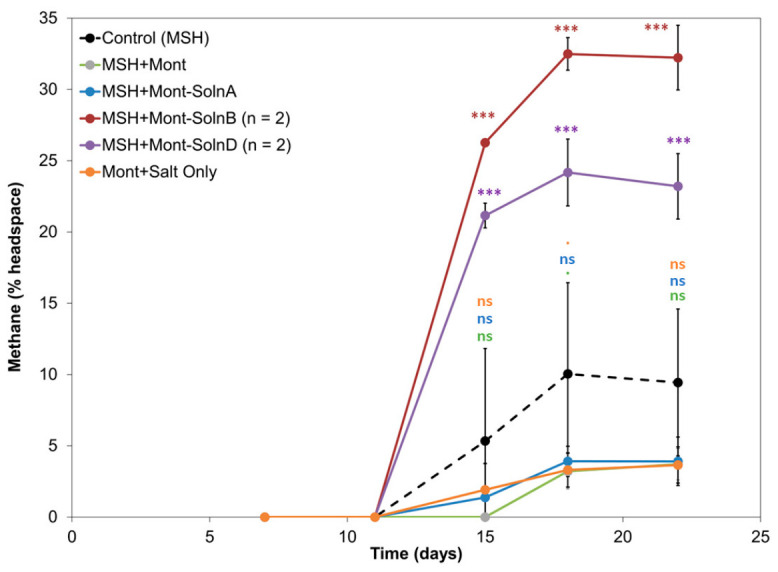
Methane production (% headspace) over time for *Methanococcus maripaludis* grown in eight variations of MSH medium at room temperature (ca. 22 °C). Conditions labeled “Mont” contain 0.5 g montmorillonite. Components of Solutions A, B, and D (SolnA, SolnB, SolnD, respectively) and the salt solution (“Salt”) are given in Table 4. Data are not shown for conditions under which no methane was produced by any replicates (MSH + Mont-Salt [MSH medium containing 0.5 g montmorillonite and no salt solution] and Buffer + Mont [bicarbonate buffer containing 0.5 g montmorillonite only]). Control tubes (black dashed line) refer to cultures of *M. maripaludis* grown in standard MSH medium. Data are the average of three biological replicates unless otherwise indicated. Error bars represent ± one standard deviation. Statistical differences revealed by ANOVA are presented compared to the control condition (ns: not significant, .: *p* < 0.1, ***: *p* < 0.001).

The highest CH_4_ concentration by *M. maripaludis* was achieved after 18 days’ incubation at 22 °C (23.9 ± 14.8% headspace) in cultures containing MSH medium with 0.5 g montmorillonite but without Solution B (200 g/L K_2_PO_4_·3H_2_O; *p* < 0.001 (compared to control tubes), Table 4, Figure 5). Cultures containing MSH medium and 0.5 g montmorillonite, but without Solution D (trace minerals; Table 4), achieved an average maximum CH_4_ concentration of 19.5 ± 8.3% headspace, also after 18 days’ incubation (*p* < 0.001, Table 4, Figure 5). Methane production in MSH medium alone (control samples) also peaked at 18 days’ incubation, reaching 9.4 ± 5.2% headspace. Methane production in the cultures containing MSH with 0.5 g montmorillonite without Solution A or solely 0.5 g montmorillonite and the salt solution (per L, 1.475 g NaCl, 0.085 g MgCl_2_, and 0.025 g KCl; Table 4) reached similar CH_4_ concentrations near 3–4% headspace after 18–22 days’ incubation and were not significantly different from the CH_4_ concentration in the control tubes (*p* > 0.1, Figure 5). Cultures grown in solutions consisting of MSH medium with 0.5 g montmorillonite but without the salt solution and solely bicarbonate buffer with 0.5 g montmorillonite failed to produce any CH_4_.

## 4. Discussion

Here we tested the ability of four methanogens, often used to represent potential model organisms for life on Mars, to grow in the presence of two clay minerals (illite or nontronite) and one Martian simulant regolith (MMS) in their standard growth media. The aim was to determine if the presence of the Mars simulant would inhibit, enhance, or have no effect on CH_4_ production. Additionally, we tested the ability of these four methanogens to produce CH_4_ using nutrients available solely within the clay mineral montmorillonite, without the addition of salts or trace minerals to the bicarbonate buffer. The goal of this experiment was to determine if a Mars simulant contains the necessary nutrients to support methanogen growth given the availability of H_2_ and CO_2_ as energy and carbon sources, respectively. Overall, the results shown here indicate that each of the four methanogens was capable of growth in the presence of multiple Mars simulants, albeit in their standard methanogenic growth medium. When solely provided with H_2_, CO_2_ (in the form of bicarbonate buffer), Na_2_S, and montmorillonite, only three of the four methanogens (*M. formicicum, M. barkeri, M. wolfeii*) were capable of growth. In all, aside from a few select cases (i.e., *M. maripaludis* grown in the presence of 2% (*w*/*v*) illite or *M. maripaludis* grown in bicarbonate buffer containing 10% (*w*/*v*) montmorillonite), none of the Mars simulants proved completely biocidal to the methanogens tested, with enhanced CH_4_ production seen in certain instances. While CH_4_ production varied amongst the four species and the Mars simulants tested, variation in CH_4_ concentrations is not unusual, even amongst individual replicates [132,133]. The ability of these methanogens to metabolize in the presence of these Mars simulants adds further support to the possibility that methanogens may have existed or may still exist in a Martian subsurface environment.

*M. maripaludis* was the methanogen that was most sensitive to the addition of Mars simulant to the growth medium or buffer, with all replicates failing to grow in cultures containing the organism’s standard growth medium (MSH medium) with 2% (*w*/*v*) illite (Figure 1B) or in bicarbonate buffer containing 10% (*w*/*v*) montmorillonite (Table 3). Methane production by *M. maripaludis* was also more greatly affected by the presence of MMS than for either *M. wolfeii* or *M. formicicum,* which also saw decreases in CH_4_ production in cultures containing MMS (Figure 3). The increased sensitivity of *M. maripaludis* to the presence of Mars simulants may be due to its halophilic nature. *M. maripaludis* was originally isolated from salt marsh sediment and found to require at least 5 mM Mg for growth [161]. Jones et al. [161] also discovered that neither sodium (Na) nor calcium (Ca) could substitute for this Mg requirement. Compared to the other methanogens tested here, the cell wall of *M. maripaludis* is a single, electron-dense, proteinaceous S-layer, lacking peptidoglycan molecules, which easily lyses [161,162] and likely contributes to the sensitivity seen here. In contrast, *M. barkeri* is considered to have a relatively thick and rigid cell wall [163], but also tends to form aggregates in culture, which may aid in survival [164,165,166]. Similarly, *M. formicicum* and *M. wolfeii* are described as having thick and rigid cell walls containing pseudomurein [167,168,169], also contributing to their robustness. Lastly, the genome for *M. wolfeii* encodes for a relatively large number of glycosyltransferases, which aid in the maintenance of cell integrity and stability, and are believed to contribute to the organism’s ability to adapt to harsh environments [170].

Previous studies have also looked at the ability of methanogens to produce CH_4_ in the presence of Mars simulants including nontronite, illite, montmorillonite, and MMS [82,83,122,123]. In experiments with *Methanosarcina mazei*, Zhang et al. [82] assessed both CH_4_ production and Fe reduction in the presence of either nontronite or illite. The authors discovered that CH_4_ production on these clays after 20 days’ incubation at 37 °C was typically less than one-third the amount produced in cultures without clays (ca. 0.0015 mmol CH_4_ vs. ca. 0.0055 mmol CH_4_) and that CH_4_ inhibition was directly correlated with the extent of bioreduction [82]. Importantly, however, and in contrast to the experiments conducted here, 0.5% (*v*/*v*) methanol was added to the cultures as a carbon source with the reduction of Fe^3+^ coupled to the oxidation of methanol [82]. Similarly, Zhang et al. [83] measured both CH_4_ production and Fe reduction by *Methanothermobacter thermautotrophicus* in cultures containing either 5 g/L [0.5% (*w*/*v*)] montmorillonite or nontronite. With either clay, Zhang et al. [83] found CH_4_ production by *M. thermautotrophicus* to be significant (ca. 0.12–0.16 mmol after 40 days’ incubation at 65 °C). In comparison to the experiments conducted here, after 35 days’ incubation in cultures containing 11.1% (*w*/*v*) nontronite, *M. barkeri* produced 8.8 ± 7.4% headspace CH_4_, *M. wolfeii* produced 23.0 ± 18.1% headspace CH_4_, and *M. formicicum* produced 17.9 ± 15.3% headspace CH_4_ (Figure 2, Table 2), equating to ca. 0.052 mmol, 0.13 mmol, and 0.11 mmol CH_4_, respectively (see Appendix A for calculations). Methane production was similar for *M. wolfeii* and *M. formicicum*, compared to *M. thermautotrophicus*, despite differences in experimental methodology including the final clay concentration in the medium and how the clay was provided to the methanogens.

All four methanogens proved capable of producing CH_4_ in the presence of up to 33.3% (*w*/*v*) nontronite (Figure 2), which may not be surprising considering that nontronite is an iron (Fe) smectite, and Fe is critical for methanogenesis (as Fe/Fe or Ni/Fe clusters used by hydrogenases [102]). The lowest amount of CH_4_ produced in the presence of 33.3% (*w*/*v*) nontronite in the experiments conducted here was by *M. formicicum* (Figure 2C) and averaged 2.4 ± 1.9% headspace after 35 days’ incubation at 37 °C. This is equivalent to 0.014 mmol CH_4_ (see Appendix A for calculations), about ten times lower than the CH_4_ produced by *M. thermautotrophicus* with 0.5% (*w*/*v*) nontronite [83]. However, the greatest amount of CH_4_ produced in the presence of 33.3% (*w*/*v*) nontronite averaged 28.5 ± 7.2% headspace, which is equivalent to ca. 0.18 mmol, and was produced by *M. maripaludis* after 35 days’ incubation at 22 °C (Figure 2B). As mentioned above, the discrepancy between the amount of CH_4_ produced between the two studies likely relates to the concentration of clays within the tubes [i.e., 11–33% (*w*/*v*) vs. 0.5% (*w*/*v*)] and the mechanism of integration (e.g., creating a slurry). Additionally, the lower CH_4_ concentrations in cultures containing nontronite may be due to Fe reduction by the methanogen as suggested by Zhang et al. [83]. While direct comparison is difficult due to differences in experimental conditions (i.e., the methanogens used, the optimal growth temperatures, specific medium components, how the clay minerals were added to the medium, etc.), both data provided here and data from Zhang et al. [83] indicate that certain methanogens are capable of growth (CH_4_ production) in the presence of nontronite. Surprisingly, the greatest CH_4_ concentrations in the presence of nontronite were produced by cultures of *M. maripaludis*, which showed the highest sensitivity (inhibition of CH_4_ production) to the other Mars simulants tested.

Significant differences are seen in both CH_4_ concentration and time to maximum CH_4_ concentration amongst the three methanogens and three Mars simulants tested here and between the experiments conducted by Sinha and Kral [122], including amongst control tubes (Table S1). For control cultures, as well as samples containing montmorillonite, CH_4_ concentrations were much higher and achieved much more quickly in experiments conducted by Sinha and Kral [122] (Table S1). However, variation amongst experimental procedures could explain the differences in observed CH_4_ production. For example, Sinha and Kral [122] measured CH_4_ production from serum bottles containing 3 g Mars simulant and 60 mL [5% (*w*/*v*)] bicarbonate buffer (leaving ca. 90 mL headspace). Here, experiments took place in Balch tubes (ca. 25 mL total volume) and contained variable amounts of Mars simulant and medium. Incubation temperatures and amount of H_2_ provided (i.e., 2 bar) were the same. However, taking into account available headspace volume and converting to mmol CH_4_ (see Appendix A), CH_4_ production by *M. formicicum* was 7.8–11.4x greater in control samples as measured by Sinha and Kral [122] compared to experiments performed here (Table S1).

One major factor that might be attributable to the differences in CH_4_ production is that in neither study (Sinha and Kral [122] nor here) were inocula standardized to cell number. In Sinha and Kral [122], an unspecified volume of washed cells were resuspended in 15 mL bicarbonate buffer, and 1 mL of resuspended cells was used to inoculate each serum bottle. In contrast, the experiments conducted here typically used 0.5 mL culture as inocula (the montmorillonite experiment used 0.5 mL washed cells as the initial inoculum; Table S1). Further, for the nontronite and MMS experiments, the concentration of the Mars simulant were much higher in the experiments conducted here than in Sinha and Kral [122], which could affect CH_4_ production, although, ultimately, this does not explain the differences in CH_4_ concentration between the controls.

Chastain and Kral [123] looked at the ability of one methanogen, *M. wolfeii*, to utilize montmorillonite as a nutrient source without a reducing or buffering agent in the medium: the bicarbonate buffer was replaced with the liquid fraction of a 1% montmorillonite/deionized water suspension and no Na_2_S was added to the medium. The authors found that, compared to buffered and reduced cultures, cultures containing solely H_2_, CO_2_, montmorillonite, and the liquid fraction of a montmorillonite-in-water suspension resulted in CH_4_ production that was much slower and reached much lower concentrations (buffered/reduced: 26.2 ± 7.9% headspace CH_4_ after 21 days’ incubation vs. non-buffered/reduced: 10.2 ± 0.5% headspace CH_4_ after 96 days’ incubation) [123]. The authors attributed the lower CH_4_ concentration to ‘simple metabolism’ (non-dividing, non-growing cells), compared to conventional microbial growth (i.e., increasing cell number) [123]. Despite differences in CH_4_ production amongst these various studies, which could be attributable to differences in cell number in the inocula as discussed above, active metabolism by methanogens, as measured by CH_4_ production, is possible using some Mars simulants, such as montmorillonite, as nutrient sources, where these clays are enabling maintenance metabolism or allowing for cell division.

One factor not investigated in this study nor others mentioned here [82,83,122,123] is how the grain size, available pore space, or method of integration (of the clay mineral and the medium) affects CH_4_ production or bioreduction. For example, Zhang et al. [83] first separated the 0.02–0.5 µm fraction from each clay and then created individual slurries with final concentrations of 5 g/L [0.5% (*w*/*v*)]. These slurries were added to the methanogenic growth medium and supplemented with additional Al to promote illite formation [83]. In contrast, here, 1 g nontronite was added to tubes containing 9 mL medium, or 2.5 g nontronite was added to tubes containing 7.5 mL medium, resulting in much higher concentrations [11.1% and 33.3% (*w*/*v*)], although the static incubation of these cultures and the limited dispersal mechanism (i.e., no mixing of the clay and medium) may have reduced any negative impact that the presence of the clay may have had on the methanogens. More specifically, the slurry produced by Zhang et al. [83] likely resulted in more clay substrate being made available to the methanogenic population, which could either be beneficial in providing a substrate for metabolism (and/or bioreduction) and/or could have inhibited CH_4_ production. While bioreduction was not measured here, results from Zhang et al. [82] and Zhang et al. [83] suggest that the intricacies of microbial metabolism (methanogenesis) and Fe reduction may serve to complicate the assessment of CH_4_ production in the presence of Fe-containing Mars simulants. The correlation between inhibition of CH_4_ production and Fe reduction could also explain the inhibition of CH_4_ production for certain methanogens as seen in many of the experiments conducted here; additional experiments would be necessary to confirm this but could be an interesting avenue for future experiments.

It is important to note that Cervini-Silva et al. [171] conducted abiotic experiments in which they reacted nontronite with bicarbonate buffer and measured subsequent CH_4_ production. The highest CH_4_ concentration reached was 2040 ppbv (ca. 1.3 nmol CH_4_; see Appendix A for calculations) after 60 days’ incubation at 25 °C. However, the authors also performed scanning electron microscopy (SEM) and noted the presence of hollow vesicles within the nontronite and attributed these to evidence of Fe^2+^-oxidizing bacteria, as well as to evidence of gas trapping [171], which suggests that the experiments may not have been truly abiotic. On the other hand, the presence of hollow vesicles could also be indicative of abiotic mineralogical processes [172]. Regardless, the CH_4_ concentration measured by Cervini-Silva et al. [171] (ca. 1.3 nmol CH_4_) is well below that which we reliably consider to be the result of biotic methane production (i.e., ca. 0.1% headspace or ca. 0.61 µmol CH_4_; see Appendix A, Chastain and Kral [123]). Thus, we can confidently attribute the CH_4_ production shown in Figure 2 to methanogenesis and not to the reaction between nontronite and bicarbonate in the tubes.

Differences in experimental procedures limit the ability to directly compare the extent of the effects that these Mars simulants have on CH_4_ production by methanogens. However, data provided here serve to confirm that while CH_4_ production may be reduced in the presence of Mars simulants, these components are not completely inhibitory or biocidal for certain methanogens. Furthermore, as evidenced by both the montmorillonite experiments and the ‘minimal medium requirements’ experiment conducted here, Mars simulants may be capable of providing certain nutrients to support or enhance microbial growth. For example, CH_4_ production was higher for *M. barkeri* in standard methanogenic growth medium in the presence of MMS than without (after 52 days’ growth, *p* < 0.01, Figure 3). In addition, CH_4_ production was significantly higher (*p* < 0.001) for *M. maripaludis* cultures containing standard methanogenic growth medium without 200 g/L K_2_PO_4_·3H_2_O (Solution B) or without trace minerals (Solution D), but with 0.5 g montmorillonite (Table 4, Figure 5). With evidence for a warm and wet early Mars provided by surface weathering of clays under acidic conditions [89,90,92], leached minerals could have provided sufficient nutrients for microbial life on the planet and could potentially explain certain results seen here. While elemental concentrations within leachates were not measured here, the availability of certain minerals leached from the clays over time could explain the increased CH_4_ production by *M. barkeri* in the presence of MMS, if the methanogen was limited for a particular trace mineral within its growth medium that was available within the Mars simulant. Similarly, for *M. maripaludis* in cultures containing montmorillonite but without K_2_PO_4_ or trace minerals, the organism likely was capable of utilizing trace minerals available directly from the montmorillonite itself. For example, in an earlier work, Altheide et al. [86] conducted acidic weathering reactions using both nontronite and montmorillonite and measured elemental concentrations within the resultant leachate. For nontronite, the authors discovered that, generally, elemental concentration within the leachate significantly increased with decreasing pH of the initial solution. In particular, elemental concentrations of Ti, Fe, Ca, K, Al, Mg, and Na all increased from concentrations below 1 mg/L to values ranging from 63 mg/L (K) to over 18,600 mg/L (Fe). Similarly, for montmorillonite, while only leachate from weathering reactions using a solution with pH 0 was tested, concentrations of Ti, Fe, Ca, K, Al, and Mg also increased, though concentrations were much lower than those for nontronite (ca. 17–1330 mg/L) and no Na was present (<0.01 mg/L) [86]. Importantly, however, media used here were not expected to be acidic with typical pH measurements (in the absence of Mars simulants) near 6.6–7.0 [151,152,173].

Future studies could further explore optimal and minimal medium requirements through additional experiments with and without various concentrations of salts, trace minerals, and other medium components. The use of standard inocula, either through cell counts, optical density values, or dry cell weight (DCW) measurements would better enable comparison to previous experiments. It would also serve future studies to include cell counts or DCW, of both inocula and samples taken during the length of the experiment, which could delineate between maintenance metabolism and cell division. For experiments utilizing Mars simulants as nutrient sources, experiments should include elemental measurements within the simulants themselves as well as the leachates, as previously performed under abiotic conditions by Altheide et al. [86]. Additional data such as gene expression, as collected for *M. barkeri* by Harris et al. [126] and Harris and Schuerger [120], would also be extremely informative regarding stress tolerances and metabolism, but were outside the scope of the studies conducted here. Ultimately, the data shown here provide support for the possibility of past or present microbial life on Mars.

## 5. Conclusions

While these experiments did not aim to replicate current or past conditions on Mars, the ability of these four methanogens to grow in the presence of multiple Mars simulants suggests that certain components within Martian regolith are not inherently biocidal, albeit, given the presence of abundant carbon (i.e., CO_2_) and energy (i.e., H_2_) sources, as well as additional nutrients, liquid water, and satisfactory growth temperatures. Furthermore, the ability of *M. wolfeii*, *M. barkeri*, and *M. formicicum* to produce CH_4_ in cultures containing solely bicarbonate buffer, H_2_ gas, Na_2_S, and montmorillonite, indicates that Martian regolith, potentially, could have provided vital nutrients, likely present as interlayer cations, such as K^+^, Na^+^, and Ca^2+^, for past life or could potentially currently support extant microbial metabolism(s) in the Martian subsurface; although, additional experiments reflecting past or present Martian subsurface pressures and temperatures, as well as realistic water activity regimes, are required to support this statement. It is also important to note that while methanogens are obligate CH_4_ producers, increasing CH_4_ concentrations over time may only be indicative of maintenance metabolism and not correspond to dividing cells. However, we argue that active metabolism, with specific respect to aeonophiles, remains relevant to both terrestrial and Martian deep subsurface microbial communities, if present.

Overall, there were varied results among replicates and across species and Mars simulants; however, the actual CH_4_ concentrations are less important than the fact that CH_4_ was or was not produced. Future experiments including analyses such as cell counts or biomass measurements in terms of DCW may better enable more accurate comparisons against previous studies. In the context of microbial–clay interactions, measuring the concentration of elements in solution or the cation exchange capacity of the Mars simulants over time would further inform the effect of these simulants on microbial metabolism. Additionally, the potential for bioreduction of Mars simulants, while also inhibiting CH_4_ production, poses a unique opportunity to further explore the possibility of the existence of methanogenic Archaea on Fe-rich Mars. While much more labor- and analysis-intensive, transcriptomics and/or proteomics approaches have been useful in more recent methanogen studies and could also help to elucidate metabolic effects of the Mars simulants.

## Figures and Tables

**Table 1:** Mars simulant physical and chemical data ^1^.

Clay	Nontronite ^2, 3^	Illite ^2, 3^	Montmorillonite ^2, 3, 4^	Mojave Mars Simulant ^5^
**Type**	Iron smectite	Aluminum phyllosilicate	Aluminum smectite	Martian regolith simulant
**Identifier**	NAu-1	IMt-1/2	SWy-1/2/3	MMS
**Origin**	Uley, Australia	Silver Hill, MT, USA	Crook County, WY, USA	Mojave Desert, CA, USA
SiO_2_	53.33	49.3	62.9	49.4
Fe_2_O_3_	34.19	7.32	3.35	10.87
Al_2_O_3_	10.22	24.25	19.6	17.1
CaO	3.47	0.43	1.68	10.45
MgO	0.27	2.56	3.05	6.08
Na_2_O	0.08	0	1.53	3.28
K_2_O	0.03	7.83	0.53	0.48
TiO_2_		0.55	0.090	1.09
P_2_O_5_		0.08	0.049	0.17
MnO		0.03	0.006	0.17
FeO		0.55	0.32	
F			0.111	
S			0.05	
SO_3_				0.10
Cr_2_O_3_				0.05

^1^ Chemical compositions are given in percent (%). ^2^ Data from van Olphen and Fripiar [159] and available from clays.org/sourceclays_data/. ^3^ Data is unofficial and is meant to be used as a guideline and not as an analytical certification. ^4^ Chemical composition not available from Ward’s Science. Example chemical components for montmorillonite sourced from Crook County, WY, USA given from ^2^. ^5^ General chemical composition from Peters et al. [149].

**Table 2:** Experimental conditions and maximum methane concentrations produced by *Methanobacterium formicicum*, *Methanosarcina barkeri*, *Methanothermobacter wolfeii*, and *Methanococcus maripaludis* grown in the presence of Mars simulants in standard methanogenic growth media.

Experiment	Organism	Clay Concentration (% *w*/*v*)	Clay Concentration (g Added/mL Culture)	Number of Replicates	Total Length of Incubation (Days)	Length of Incubation to Maximum Methane Concentration (Days)	Maximum Methane Concentration (% Headspace)
Illite	*M. formicicum*	0	0/10	4	31	31	7.4 ± 0.5
		1	0.1/10	4		16	24.6 ± 5.2
		2	0.2/10	3			26.7 ± 2.6
Illite	*M. barkeri*	0	0/10	5	31	31	1.2 ± 0.3
		1	0.1/10	4			1.1 ± 0.3
		2	0.2/10	5			1.4 ± 0.5
Illite	*M. wolfeii*	0	0/10	4	31	24	22.5 ± 1.0
		1	0.1/10	3	29	29	29.6 ± 2.8
		2	0.2/10	3	31	16	24.9 ± 0.6
Illite	*M. maripaludis*	0	0/10	4	31	31	12.7 ± 3.2
		1	0.1/10	5			13.6 ± 6.0
		2	0.2/10	4			0
Nontronite	*M. formicicum*	0	0/10	3	35	35	44.3 ± 3.4
		11.1	1/9				17.9 ± 15.3
		33.3	2.5/7.5				2.4 ± 1.9
Nontronite	*M. barkeri*	0	0/10	3	35	35	16.1 ± 3.9
		11.1	1/9				8.8 ± 7.4
		33.3	2.5/7.5				13.3 ± 14.7
Nontronite	*M. wolfeii*	0	0/10	3	35	35	41.0 ± 3.0
		11.1	1/9				23.0 ± 18.1
		33.3	2.5/7.5				12.1 ± 19.4
Nontronite	*M. maripaludis*	0	0/10	3	35	35	42.3 ± 6.6
		11.1	1/9				26.3 ± 14.8
		33.3	2.5/7.5				28.5 ± 7.2
Mojave Mars Simulant	*M. formicicum*	0	0/10	2	140	52	29.9 ± 0.7
		100	10/10	3			25.9 ± 1.5
Mojave Mars Simulant	*M. barkeri*	0	0/10	2	140	52	4.8 ± 1.6
		100	10/10	3			15.1 ± 1.0
Mojave Mars Simulant	*M. wolfeii*	0	0/10	2	140	9	26.8 ± 2.1
		100	10/10	3		52	20.9 ± 1.7
Mojave Mars Simulant	*M. maripaludis*	0	0/10	2	140	24	28.6 ± 0.2
		100	10/10	3			8.4 ± 10.4

**Table 3:** Experimental conditions and maximum methane concentrations produced by *Methanobacterium formicicum*, *Methanosarcina barkeri*, *Methanothermobacter wolfeii*, and *Methanococcus maripaludis* grown in bicarbonate buffer containing molecular hydrogen, sodium sulfide, and montmorillonite.

Experiment	Organism	Clay Concentration (% *w*/*v*)	Clay Concentration (g Added/mL Buffer)	Number of Replicates	Total Length of Incubation (Days)	Length of Incubation to Maximum Methane Concentration (Days)	Maximum Methane Concentration (% Headspace)
Montmorillonite	*M. formicicum*	10 (Set A)	1/10	3	198	42	14.8 ± 4.0
		10 (Set B)			156	156	39.1 ± 10.1
		10 (Set C)			114	114	11.7 ± 11.7
Montmorillonite	*M. barkeri*	10 (Set A)	1/10	3	198	84	7.8 ± 1.0
		10 (Set B)			156	156	18.5 ± 8.6
		10 (Set C)			114	114	19.0 ± 6.0
Montmorillonite	*M. wolfeii*	10 (Set A)	1/10	3	198	84	23.5 ± 19.4
		10 (Set B)			156	156	49.3 ± 25.3
		10 (Set C)			114	114	49.3 ± 4.4
Montmorillonite	*M. maripaludis*	10 (Set A)	1/10	3	198	198	0
		10 (Set B)			156	156	0
		10 (Set C)			114	114	0

**Table 4:** Components for eight variations of MSH medium with and without montmorillonite.

Per 100 mL	Control (MSH Medium)	Mont ^1^ + Salt	MSH + Mont-SolnA	MSH + Mont-SolnB	MSH + Mont-SolnD	MSH + Mont-Salt	Mont + Buffer ^2^ Only	Mont + MSH
0.5 g montmorillonite (per 10 mL)	-- ^3^	X ^4^	X	X	X	X	X	X
Solution A ^5^, 500 µL	X	--	--	X	X	X	--	X
Solution B ^6^, 100 µL	X	--	X	--	X	X	--	X
Solution C ^7^, 100 µL	X	--	X	X	X	X	--	X
Solution D ^8^, 50 µL	X	--	X	X	--	X	--	X
0.1 g yeast extract	X	--	X	X	X	X	--	X
0.1 g trypticase peptone	X	--	X	X	X	X	--	X
0.025 g mercaptoethane sulfonic acid	X	--	X	X	X	X	--	X
1.475 g NaCl	X	X	X	X	X	--	--	X
0.085 g MgCl_2_	X	X	X	X	X	--	--	X
0.025 g KCl	X	X	X	X	X	--	--	X
Buffer ^5^	X	X	X	X	X	X	X	X

^1^ Mont = montmorillonite. ^2^ Bicarbonate buffer: 4 g/L NaOH saturated with CO_2_. ^3^ “--” denotes that this ingredient is absent from the medium. ^4^ “X” denotes that this ingredient is present in the medium. ^5^ Solution A, per L: 100 g NH_4_Cl, 100 g MgCl_2_·6H_2_O, 40 g CaCl_2_·2H_2_O. ^6^ Solution B, per L: 200 g K_2_PO_4_·3H_2_O. ^7^ Solution C, per L: 0.5 g resazurin. ^8^ Solution D, per L: 500 mg Na_2_-EDTA·2H_2_O, 150 mg CoCl_2_·6H_2_O, 100 mg MnCl_2_·4H_2_O, 100 mg FeSO_4_·7H_2_O, 100 mg ZnCl_2_, 40 mg AlCl_3_·6H_2_O, 30 mg Na_2_WO_4_·2H_2_O, 20 mg CuCl_2_·2H_2_O, 20 mg NiSO_4_·6H_2_O, 10 mg H_2_SeO_3_, 10 mg H_3_BO_3_, 10 mg Na_2_MoO_4_·2H_2_O.

## Data Availability

Data available upon request.

## Supplementary Materials

The following supporting information can be downloaded at https://www.mdpi.com/article/10.3390/microorganisms14071496/s1, Table S1: Maximum methane concentrations (% headspace) for *Methanothermobacter wolfeii*, *Methanosarcina barkeri*, and *Methanobacterium formicicum* grown on Mars simulants.

## Abbreviations

The following abbreviations are used in this manuscript:

Al	Aluminum
ATCC	American Type Culture Collection
C	Carbon
Ca	Calcium
CH_4_	Methane
CO_2_	Carbon dioxide
Cr	Chromium
Cu	Copper
Fe	Iron
H_2_	Hydrogen
H_3_O^+^	Hydronium
K	Potassium
Li	Lithium
Mg	Magnesium
Mn	Manganese
MMS	Mojave Mars Simulant
N	Nitrogen
Na	Sodium
NaOH	Sodium hydroxide
Na_2_S	Sodium sulfide
NH_4_	Ammonium chloride
OCM	Oregon Collection of Methanogens
P	Phosphorus
S	Sulfur
SEM	Scanning electron microscopy
Si	Silicon
Ti	Titanium
Zn	Zinc

## Appendix A

### Appendix A.1. Calculations Converting Methane from % Headspace to mol

For comparison to methane (CH_4_) production measured in millimoles (mmol) by Zhang et al. [82] and Zhang et al. [83], we have converted our headspace CH_4_ data to moles (mol) using the ideal gas law (A1) and the following conditions:

(A1) $$n\;\left(moles\;{CH}_{4}\right)=\frac{PV}{RT}$$

where *P* = pressure = 1 atm, *V* = the CH_4_ percentage of the total internal headspace volume of the culture tube, *T* = temperature, and *R* is the ideal gas constant (0.0821 L-atm/mol-K). The total internal headspace volume of the culture tube is estimated to be 0.015 L given that the Balch tubes used in all experiments conducted here have a total internal volume of 0.025 L and are filled with ca. 0.01 L medium (except the Mojave Mars Simulant (MMS) experiment; see below). Additionally, although all CH_4_ measurements were taken at room temperature (22 °C), the tubes were incubated at the organisms’ respective ideal growth temperatures (22 °C, 37 °C, or 55 °C). These incubation temperatures were used to calculate moles CH_4_ so as not to artificially inflate any concentrations. Thus, for moles CH_4_ calculations for *M. maripaludis* and *M. wolfeii*, temperatures of 295 K (22 °C) and 328 K (55 °C), respectively, were used. For *M. barkeri* and *M. formicicum*, a temperature of 310 K (37 °C) was used. For example, for a % headspace concentration of 8.8% CH_4_ produced by *M. barkeri* at 37 °C, *V* = 0.088 × 0.015 L = 0.00132 L and *T* = 310 K, equating to 0.052 mmol CH_4_. For comparison, using a temperature of 22 °C (295 K) equates to a CH_4_ concentration of 0.055 mmol.

When available headspace volume is reduced, for example, in the MMS experiments compared to the other experiments performed here, the resultant moles CH_4_ will also be fewer. More specifically, as noted above and in the Materials and Methods, the available headspace within the Balch tubes used for most of the experiments conducted here was ca. 15 mL. However, the MMS experiment contained significantly more Mars simulant than the other experiments, leaving a total available headspace ca. 10 mL. When converting to moles CH_4_ using Equation (A1), the reduced volume will correspond to fewer moles CH_4_. In particular, for a % headspace concentration of 30% by *M. formicicum* (incubation temperature: 310 K) with a total available headspace volume of 15 mL (0.015 L), Equation (A1) gives the CH_4_ concentration as 177 mmol CH_4_. For the same % headspace concentration (30%) but with a reduced headspace volume (0.01 L), Equation (A1) gives the CH_4_ concentration as 118 mmol CH_4_.

Chastain and Kral [123] measured 0.14 ± 0.05% headspace CH_4_ from *Methanothermobacter wolfeii* cultures containing carbon dioxide (CO_2_), 1 g montmorillonite, and 6 mL buffer (derived from montmorillonite and deionized water), but without an energy source [i.e., hydrogen (H_2_)]. The authors thus considered headspace concentrations greater than 0.1% to be the result of methanogenesis. Using Equation (A1), this value corresponds to 0.61 µmol CH_4_ in the experiments performed here, which we also consider to be the lower limit to attribute increases in CH_4_ concentration to active CH_4_ production by methanogens. However, as mentioned within the Materials and Methods section, we recognize that, without additional biomass data, we cannot ascertain whether this CH_4_ production corresponds to cell division or simply to maintenance metabolism. Although, we do argue that active metabolism, regardless of cell division, remains relevant to both terrestrial and Martian subsurface habitats [38,156].

### Appendix A.2. Calculations Converting Methane from ppbv to mol

Cervini-Silva et al. [171] conducted abiotic experiments in which they reacted nontronite with bicarbonate buffer and measured subsequent CH_4_ production. The highest CH_4_ concentration reached was 2040 ppbv after 60 days’ incubation at 25 °C. These experiments were conducted in 25 mL bottles and contained 100 mg nontronite in 10 mL 1 mM sodium bicarbonate (NaHCO_3_) with a nitrogen (N_2_) headspace. Assuming 0.015 L total headspace within the bottles, the total amount of N_2_ can be found using Equation (A1): with *V* = 0.015 L, *P* = 1 atm and *T* = 298 K, the total number of moles N_2_ in the headspace of the bottles is 0.61 mmol. The concentration of CH_4_ measured by Cervini-Silva et al. [160] (2040 ppbv CH_4_) equates to a 2.04 × 10^−6^ mole fraction. This equates to a CH_4_ concentration of 1.3 nmol, which is significantly lower than the value of 0.61 µmol CH_4_ that we attribute to active methanogenesis.
