# Supplementary material for: Methane Production on Mars-Relevant Clay Minerals and Simulant Regolith
Source: Microorganisms. 2026 Jul 8;14(7):1496. doi: 10.3390/microorganisms14071496 (PMC13414335; doi:10.3390/microorganisms14071496)
Supplement: Supplementary file 1 [file microorganisms-14-01496-s001.zip › microorganisms-4330079-supplementary.pdf]

microorganisms

MDPI

Article

Methane Production on Mars-Relevant Clay Minerals and Simulant Regolith

Rebecca L. Mickol [1,2,*], William Hunter Waddell [3], James Wray [3], Ryan Pohlkamp [3], Chandler Kern [3] and Timothy A. Kral [2,3,*]

1 US Naval Research Laboratory, 4555 Overlook Ave SW, Washington, DC 20375, USA
2 Arkansas Center for Space and Planetary Science, University of Arkansas, 332 N. Arkansas Ave, Fayetteville, AR 72701, USA
3 Department of Biological Sciences, University of Arkansas, Science and Engineering Room 601, Fayetteville, AR 72701, USA
* Correspondence: rebecca.l.mickol.civ@us.navy.mil (R.L.M.); tkral@uark.edu (T.A.K.)

Supplemental Information

Table S1. Maximum methane concentrations (% headspace) for *Methanothermobacter wolfeii*, *Methanosarcina barkeri*, and *Methanobacterium formicicum* grown on Mars simulants.

	Maximum Methane (mmol CH_4)[1]		Incubation Time to Maximum Methane (Days)		% (*w*/*v*) Simulant Regolith		Medium Type		Inoculum	
	SK[2]	This study[3]	SK	This study	SK	This study	SK	This study	SK	This study
**Control**[2]										
M. wolfeii	1.77	0.15 / 0.23	10	9 / 35	0	0	MM	MM	1 mL washed cells	0.5 mL active culture
M. barkeri	1.77	0.03 / 0.09	35	52 / 35	0	0	MS	MS		
M. formicicum	2.02	0.18 / 0.26	10	52 / 35	0	0	MSF	MSF		
**Montmorillonite**										
M. wolfeii	1.50	0.133	10	84	5	10	BB[4]	BB	1 mL washed cells	0.5 mL washed cells
M. barkeri	1.41	0.047	65	84	5	10	BB	BB		
M. formicicum	1.59	0.088	30	42	5	10	BB	BB		
**Mojave Mars Simulant (MMS)**										
M. wolfeii	0.267	0.078	10	52	5	100	BB	MM	1 mL washed cells	0.5 mL active culture
M. barkeri	0.177	0.059	65	52	5	100	BB	MS		
M. formicicum	0	0.102	-	52	5	100	BB	MSF		

[1]Millimoles CH_4 were calculated using the ideal gas law (see Equation (A1) in Appendix A) and assuming 90 mL available headspace in samples from Sinha and Kral [1] and 15 mL available headspace (control, montmorillonite experiments) or 10 mL available headspace (MMS experiments) in the experiments conducted here. Temperatures used for calculations were the organisms' growth temperatures: 55 °C (328 K), *M. wolfeii*; 37 °C (310 K), *M. formicicum* and *M. barkeri*. [2]SK = Sinha and Kral [1]. [3]Two examples of methane production in control tubes are shown from experiments conducted here to highlight variability in methane production amongst methanogenic cultures. The lefthand values correspond to control tubes monitored alongside the Mojave Mars Simulant (MMS) experiment shown in Figure 3 and the righthand values correspond to control tubes monitored alongside the nontronite experiment shown in Figure 2. [4]BB = bicarbonate buffer.

 https://doi.org/10.3390/microorganisms14071496

Reference

1. Sinha, N.; Kral, T.A. Stable carbon isotope fractionation by methanogens growing on different Mars regolith analogs. *Planet. Space Sci*. **2015**, *112*, 35-41. https://doi.org/10.1016/j.pss.2015.04.011.

Disclaimer/Publisher's Note: The statements, opinions and data contained in all publications are solely those of the individual author(s) and contributor(s) and not of MDPI and/or the editor(s). MDPI and/or the editor(s) disclaim responsibility for any injury to people or property resulting from any ideas, methods, instructions or products referred to in the content.

https://doi.org/10.3390/microorganisms14071496
